# What is the stable internal fixation for the unstable and osteoporotic supracondylar femoral fractures: a finite element analysis

**DOI:** 10.1186/s13018-023-04256-9

**Published:** 2023-10-07

**Authors:** Jianwei Rao, Junchao Zhang, Zhou Ye, Liguang Zhang, Jiangbao Xu

**Affiliations:** 1 The Quzhou Affiliated Hospital of Wenzhou Medical University, Quzhou, People’s Hospital, Quzhou, 324000 China; 2 Jiangshan People’s Hospital, Jiangshan, 324100 China

**Keywords:** UOSFF, ULLP, BLLP, Biomechanical study

## Abstract

**Background:**

Osteoporotic supracondylar femoral fractures (OSFF) have historically been managed by the lateral anatomical locking plate with reasonable success. However, for some kinds of unstable and osteoporotic supracondylar femoral fractures (UOSFF), especially with bone defects, unilateral locking plate (ULLP) fixation failed or resulted in implant breakage. This paper is going to explore what is the stable internal fixation for UOSFF by adding the bilateral locking plate (BLLP) fixation.

**Methods:**

OSFF models were divided into two groups according to the fracture line type, which would be further subdivided according to their angle of fracture line, presence of bone defect, location, and degree of bone defect. Thereafter, kinds of locking plate fixation were constructed. A 2010-N load was applied to the femoral head, and a 1086-N load was applied to the greater trochanter. In this condition, the maximum von Mises stress distribution of models were investigated.

**Results:**

Firstly, it was obviously found that the stress concentration in the BLLP group was more dispersed than that in the ULLP group. Secondly, according to the fracture line analysis, the stress value of fracture line type in “\” model group was higher than that of “/” model group. Moreover, with the increase in fracture line angle, the stress value of the model increased. Thirdly, from the bone defect analysis, the stress value of the medial bone defect (MBD) model group was higher than that of the lateral bone defect (LBD) model group. And as the degree of bone defect increased, the stress value increased gradually in the model group.

**Conclusion:**

In the following four cases, lateral unilateral locking plate fixation cannot effectively stabilize the fracture end, and double locking plate internal fixation is a necessary choice. First, when the angle of the fracture line is large (30, 45). Second, when the fracture line type is “/.” Third, when the bone defect is large. Fourth, when the bone defect is medial.

## Introduction

Over the past 20 years, OSFF mainly caused by low-energy trauma has recorded an increase in incidence in the elderly population [1–8]. Only osteosynthetic reconstruction with intramedullary nails or extramedullary plates can stabilize these fractures against forces and strains on the femur [9]. Intramedullary nail can provide greater stiffness but is limited by its torsion control. Due to the promotion and maturation of the MiPPO technique, plates have gradually become mainstream [10]. However, some clinical studies have shown that ULLP fixation fails or results in implant breakage when the fracture is accompanied by the severe comminution, bone loss, or osteopenia due to the thin cortex, poor bone quality, and limited bone stock available for screw purchase [11]. It has been reported that nonunion of the distal femoral after ULLP treatment occurs in 10%–23% of cases [12], and the adding of an medial locking plate is effective in preventing nonunion [13–15].

Both excessive stress values at the support and excessive movement between the fracture blocks always appear in the failure of the ULLP internal fixation, especially in UOSFF. However, clinically quantifying three-dimension (3D) fracture-site motion and stress values remains impractical, and determining the location where the ULLP fixation failed or resulted in implant breakage is also largely limited by the inability to measure or predict fracture-site motion [16–18]. Computational modeling, especially finite element analysis (FEM), permits parametric investigation at a lower cost. Such computational analysis has been proposed as a tool to study the stress, displacement, and stiffness. Besides, this analysis can help identify potential causes of internal fixation failure of locking plates and suggest improvement schemes [19].

From what has been discussed above, adding a medial locking plate (in the BLLP fixation) becomes crucial to fix the UOSFF. Therefore, we designed this study to explore the stable internal fixation method for UOSFF and to investigate the primary causes of implant breakage.

## Materials and methods

### FE analysis

In this study, a 3D-FE model of OSFF was established, which can be modified to simulate different structures according to different clinical conditions. The model consisted of two components: the osseous anatomy and the fixation hardware. The osseous anatomy was obtained from a healthy male volunteer who met the diagnostic criteria for osteoporosis (the elastic modulus of cortical and cancellous bone decreased by 33% and 66%, respectively) [30]. The lower limb CT images were acquired using a UCT530 CT machine with 120 kV/179 mAs scanning and were segmented by Mimics 15.0 software. The fixation hardware was composed of bone plates and bone screws and made of isotropic nonlinear materials TA3 and Ti6Al4V, respectively. Its elastic modulus and Poisson’s ratio can be referred to published data [31]. In addition, the tensile curve data of yield strength are shown in Table 1.

**Table 1:** Summary of structural parameters

	Elastic modulus, MPa	Poisson's ratio	Yield strength, MPa	Tensile strength, MPa
Cortical bone of femur	11,256.0	0.3	/	/
Cancellous bone of femur	197.2	0.29	/	/
Bone plate	110,000	0.3	387	563
Screw	110,000	0.3	945	1042

### Modeling fracture types and fixation methods

**Fig. 1:**
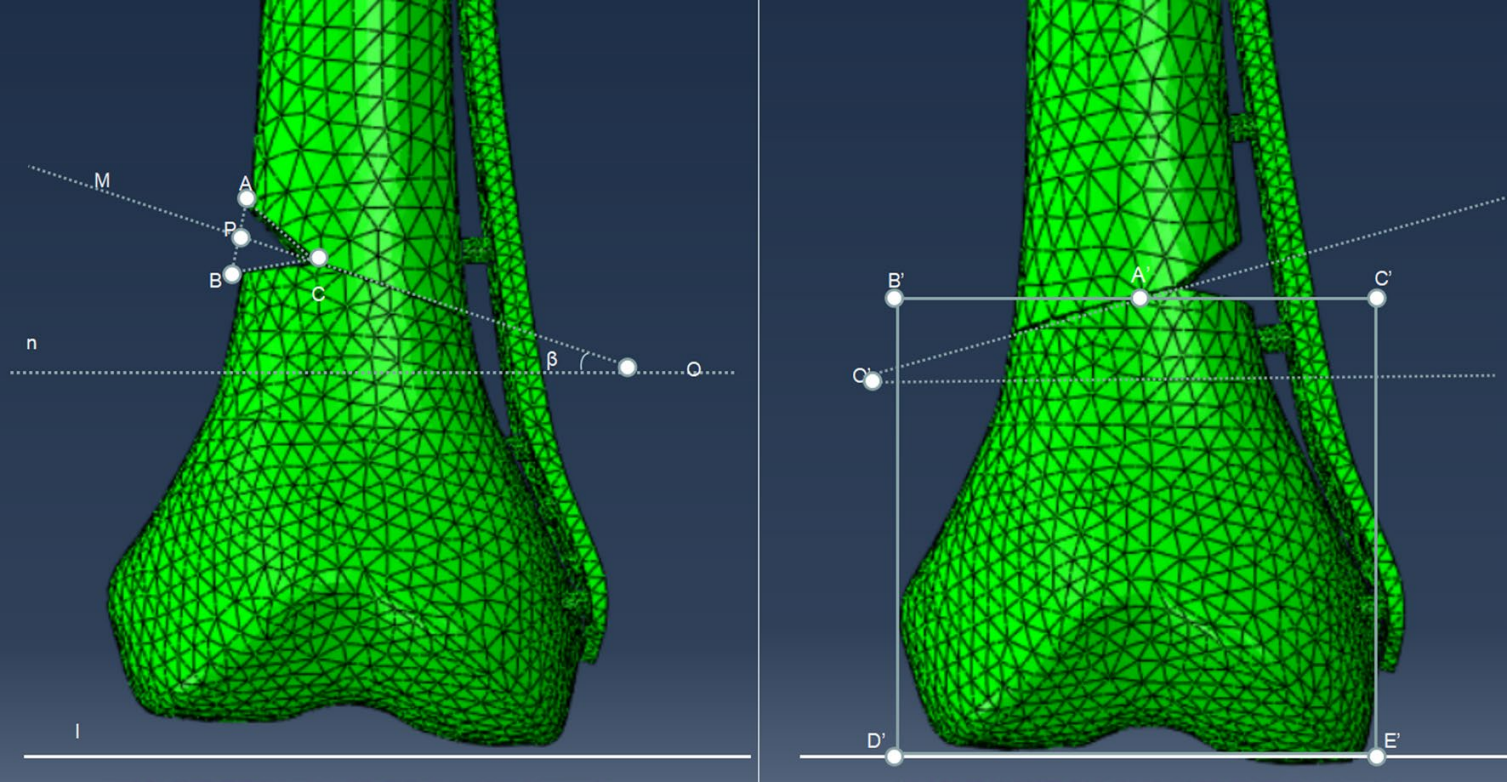
① Triangular defect: defect degree: AB = BC = AC = 5 mm, 10 mm, 15 mm, 20 mm, 30 mm, oblique fracture line angle: *β* = 15°, 30°, 45°, AP = PB. Line l was parallel to the joint surface, and line “l” and line “n” were parallel to each other. ② A′B′ = A′C′, B′D′ = B′C′ = D′E′ = C′E; the line B′C′ was the reference line of the fracture line, and the angle was adjusted with the corresponding degree at the center point A′ of the reference line

**Fig. 2:**
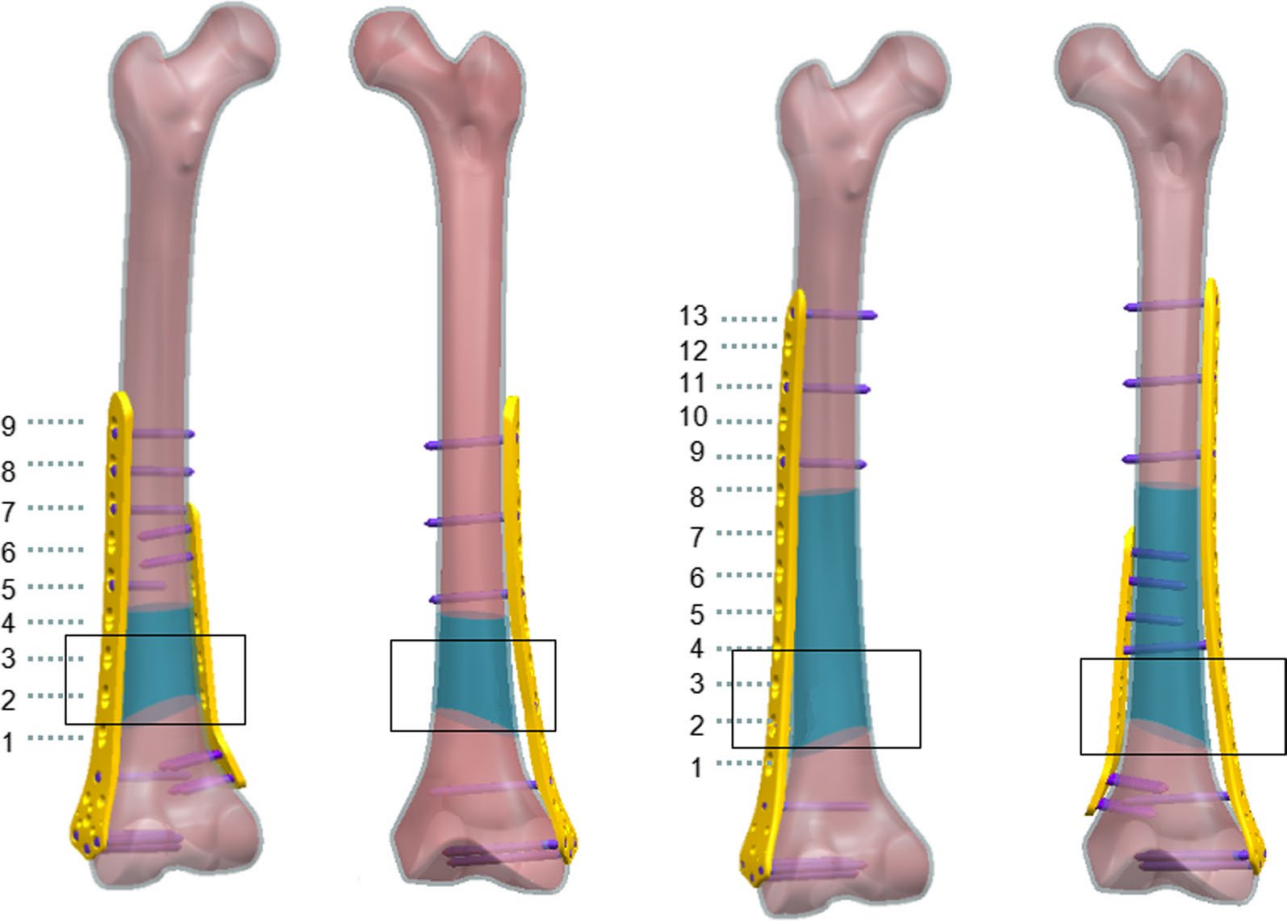
Number ① was the back view of the 9H-plate group, number ② was the front view of the 9H-plate group, number ③ was the back view of the 13H-plate group, and number ④ was the front view of the 13H-plate group. Inside the black box is the fracture line area. The blue area is a variable screw position, and stable screw positions are selected in the blue area according to the principle of minimum effective working distance [32] (not through the fracture line area)

OSFF models (OTA/AO classification of 33A or 33B) were divided into two groups according to the fracture line type (transverse fracture line or oblique fracture line). These two groups were further subdivided according to their angle of fracture line (15°/30°/45°), presence of bone defect, location of bone defect (medial/lateral), the degree of defect (5/10/15/20/30 mm), and the type of defect (triangular) as shown in Fig. 1. Thereafter, based on the engineering drawings provided by the manufacturer, we reconstructed the geometric 3D models of plates and screws. Additionally, when fixing the plates, we used either a ULLP fixation, which involved using 9-hole (9*H*) or 13-hole (13*H*), or a BLLP fixation, which involved using 9 + 6*H* or 13 + 6*H*, as shown in Fig. 2. A total of 308 models (7 * (2 * 5 + 1) * 4) were built.

### Constraints and loading conditions

**Fig. 3:**
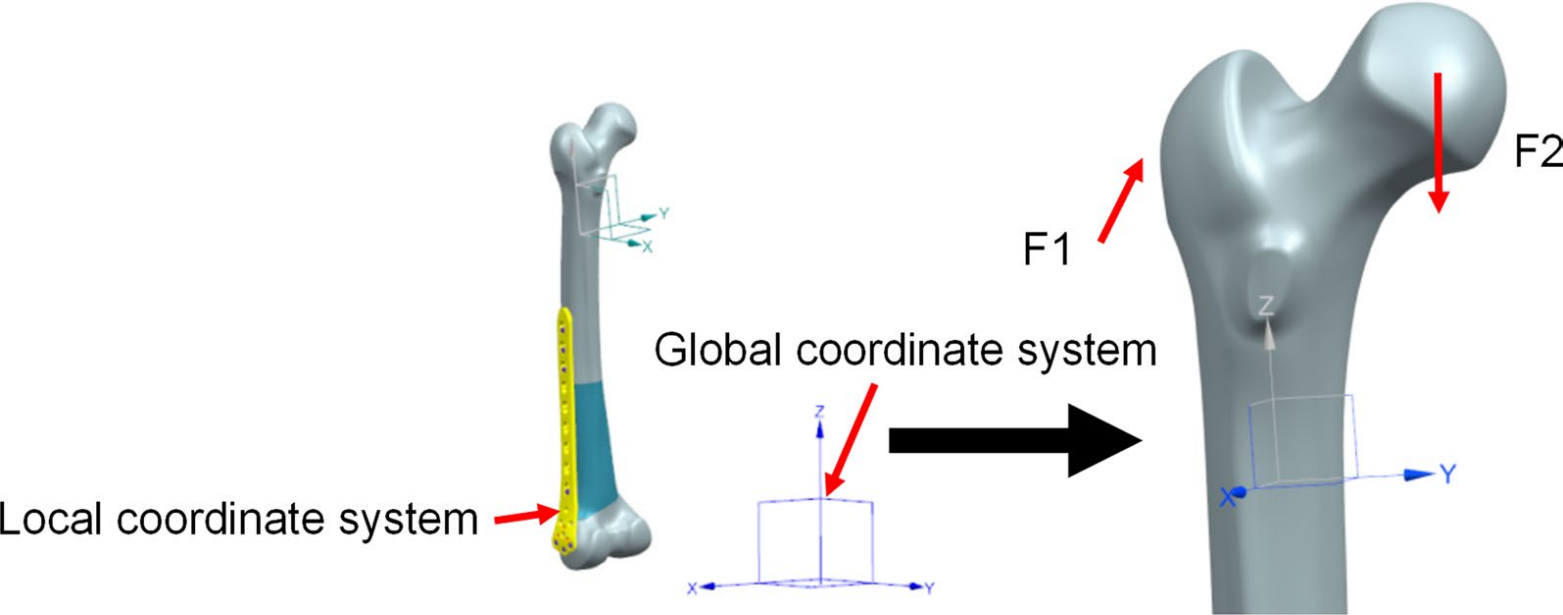
Femur loading. (Coordinate system reference standard ISO 7206-4), *F*1 is the resultant force of the muscles of the human body in the walking state [33]

The bone mesh type was C3D4 linear tetrahedral element, while the bone plate and screw mesh type was C3D10M quadratic tetrahedral element, with a total of 156,332 mesh elements. The contact surface of the fracture zone was set by friction with a friction coefficient of 0.2. Cortical bone and cancellous bone combine to form a contact surface. The connection region between the screw and the bone was set by fixed constraints. The friction method was used to set the contact surface between the plate and bone, and the friction coefficient was 0.1. According to the physiological characteristics of the hip joint, a 3D external force was applied to the proximal femur, and the global coordinate system of the 3D model was established in the upright state of the human body. A negative load of 1086 N (*F*1) was applied along the *z*-axis of the local coordinate system at the greater trochanter, and 2010 N (*F*2) was applied along the *z*-axis at the femoral head. The distal section of the femoral shaft was fully restrained [25]. The angle between *F*2 and the sagittal plane was *β* = 9°, and the angle between *F*2 and the coronal plane was *α* = 10°, as shown in Fig. 3. Subsequently, the model was imported into ABAQUS6.12-1 software to simulate the standing state of the human body.

### Validation and statistical methods of the FE model

The validation of a finite element model depends on three main aspects: (1) clinical cases (Fig. 8). (2) Previously published papers (References). (3) Whether convergence of the model can be observed. If the stress value in any part of the model is greater than a threshold, the model does not converge (stress singularity), otherwise the model converges (stress concentration). Data recording and analysis were performed using IBM® SPSS® Statistics version 22.0. And the Kolmogorov–Smirnov was used to test the assumptions of normality and homogeneity of variances. A t-test was used for normally distributed data, and non-parametric test was used for non-normally distributed data.

## Results

### Von Mises stress distribution of the femur

**Fig. 4:**
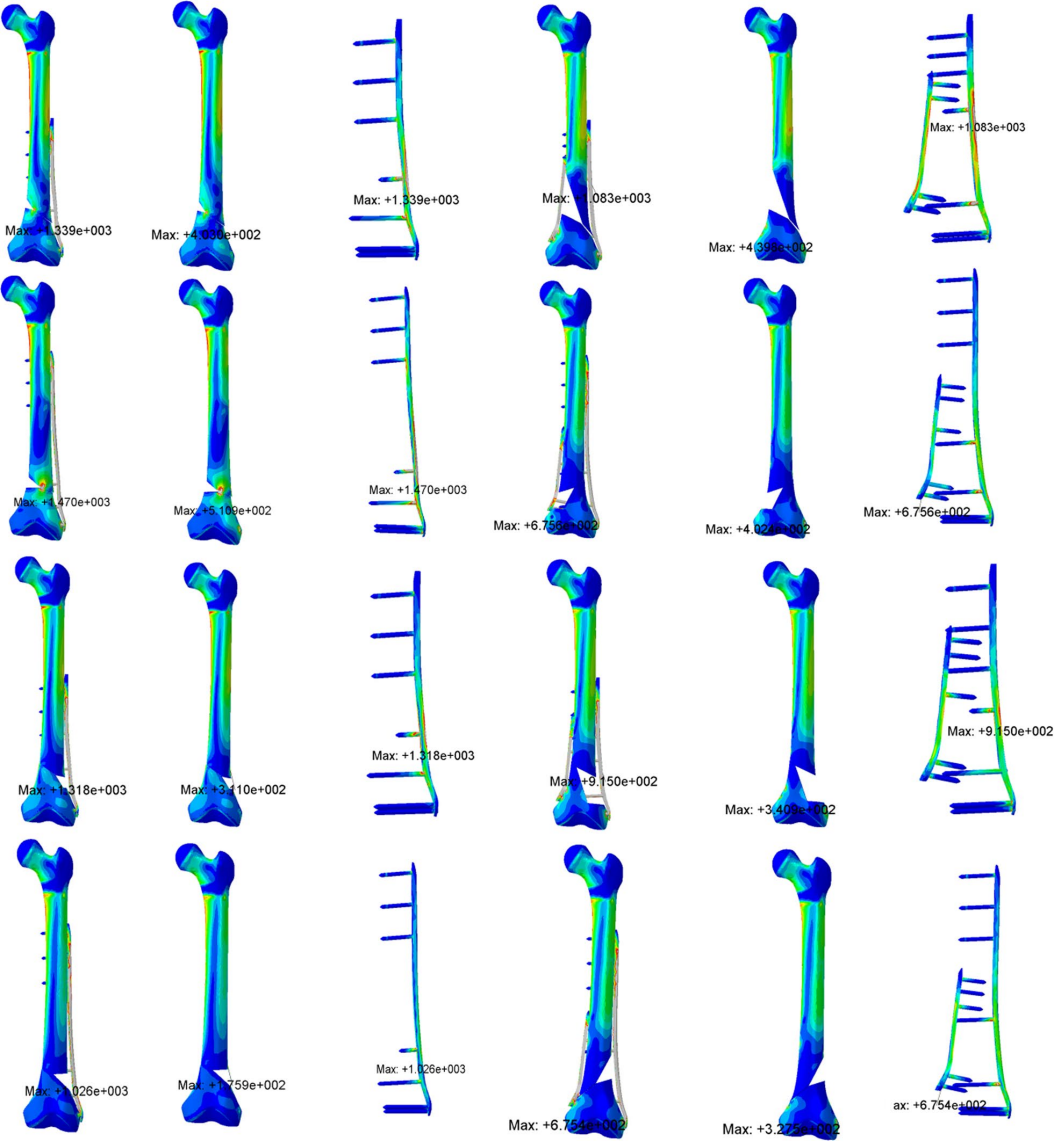
The red area represented the stress concentration

In the simulation of all models, a large stress was observed in the fracture area according to the von Mises stress distribution. Compared to the ULLP model, the maximum von Mises stress distribution at this position was much smaller for all the BLLP models (Table 2 and Fig. 4). The maximum cortical stress of the femur under the 9H and 13H ULLP models were 403 MPa and 511 MPa, respectively. Under these conditions, the cortical stress of the femoral bone under BLLP model was 296 MPa and 291 MPa, respectively. The maximum femoral stress of 9H and 13H BLLP were 440 MPa and 402 MPa, respectively (Fig. 4). Furthermore, except for the “\” model group and fracture line angle of 15°, the stress values of other models increased with the increase in fracture line angle and defect degree (Table 2).

**Table 2:** Summary of stress on femoral cortex

	Fracture line type	Defect in the medial femur							Defect in the lateral femur					
			0 mm	5 mm	10 mm	15 mm	20 mm	30 mm	0 mm	5 mm	10 mm	15 mm	20 mm	30 mm
Femoral cortical stress distribution in the case of unilateral locking plate (9H)	\	0°	124	132	168	185	269	╳	124	124	123	116	123	╳
		15°	154	215	227	284	347	╳	154	155	154	150	163	╳
		30°	288	352	403	╳	╳	╳	288	291	311	311	302	╳
		45°	╳	╳	╳	╳	╳	╳	╳	╳	╳	╳	╳	╳
	/	0°	124	132	168	185	269	╳	124	124	123	116	123	╳
		15°	119	144	151	177	296	╳	119	128	135	118	124	╳
		30°	╳	╳	╳	╳	╳	╳	╳	╳	╳	╳	╳	╳
		45°	╳	╳	╳	╳	╳	╳	╳	╳	╳	╳	╳	╳
Femoral cortical stress distribution in the case of bilateral locking plate (9H)	/	0°	170	172	178	183	207	222	170	173	179	185	178	190
		15°	203	208	213	238	246	333	203	205	219	223	213	234
		30°	277	327	296	317	314	326	277	285	278	264	282	294
		45°	306	427	410	407	413	440	306	313	293	293	307	341
	\	0°	170	172	178	183	207	222	170	173	179	185	178	190
		15°	168	175	191	177	182	227	203	187	179	180	172	266
		30°	180	197	176	187	184	211	180	171	186	187	188	255
		45°	216	220	215	218	236	╳	216	243	246	292	305	326
Femoral cortical stress distribution in the case of unilateral locking plate (13H)	/	0°	135	138	190	204	308	╳	135	122	157	120	119	╳
		15°	170	212	232	276	511	╳	170	165	155	139	176	╳
		30°	359	329	╳	╳	╳	╳	359	338	317	325	304	╳
		45°	╳	╳	╳	╳	╳	╳	╳	╳	╳	╳	╳	╳
	\	0°	135	138	190	204	308	╳	135	122	157	120	119	╳
		15°	123	148	133	185	330	╳	123	119	126	119	120	╳
		30°	╳	╳	╳	╳	╳	╳	╳	╳	╳	╳	╳	╳
		45°	╳	╳	╳	╳	╳	╳	╳	╳	╳	╳	╳	╳
Femoral cortical stress distribution in the case of bilateral locking plate (13H)	/	0°	142	154	159	177	202	276	142	146	150	168	164	167
		15°	214	239	269	278	291	304	214	218	207	214	220	221
		30°	275	308	312	318	313	323	275	289	287	302	308	307
		45°	348	359	355	356	360	356	348	360	360	352	378	373
	\	0°	142	154	159	177	202	276	142	146	150	168	164	167
		15°	148	144	147	151	161	227	148	143	148	156	160	161
		30°	150	168	171	172	180	206	150	148	165	171	173	223
		45°	277	359	370	392	402	/	277	321	327	298	328	340

*The “\” meant that the direction of fracture line was outer inferior–inner superior. The “/” meant that the direction of fracture line is outer superior–inner inferior. The “╳” meant that the model did not converge in this condition

### Von Mises stress distribution of the plates

In the simulation of all models, a large stress concentration at the fracture line was observed (Fig. 4). Compared to the ULLP model, the maximum von Mises stress distribution at this position was much smaller for all the BLLP model. The maximum stress in the long locking plate in the 9H and 13H ULLP models was 779.5 MPa and 679.4 MPa, respectively. In the 9H and 13H BLLP models, the maximum stress values of the long plate were 574.8 MPa and 577 MPa, respectively, and the maximum stress values of the short plate were 654.7 MPa and 583.8 MPa, respectively (Table 3). Furthermore, the maximum stress value was larger in the model with a larger angle to the fracture line and a larger degree of bone defect. In the BLLP model, the stress value of the long locking plate was greater than that of the small locking plate.

**Table 3:** Comparison of 9H locking plate and 13H locking plate

		Single locking plate					Double locking plate					
		FC	SC (MPa)	LP (MPa)	*X* (mm)	*Z* (mm)	FC	SC (MPa)	LP (MPa)	SP (MPa)	*X* (mm)	*Z* (mm)
9H	Min	118.6	341.2	142.6	− 4.32	− 1.70	167.7	355.1	232.1	130.7	− 4.39	− 1.75
	Median	169.9	536.2	404.4	− 5.93	− 2.22	218.3	694.4	335.5	348.6	− 5.12	− 2.38
	Mean	207.7	797.0	395.7	− 7.68	− 2.48	241.9	641.8	372	341.9	− 5.20	− 2.56
	Max	510.9	1470	779.5	− 26.56	− 6.25	439.8	1083	574.8	654.7	− 7.17	− 4.45
13H	Min	116.0	364.1	165.6	− 18.1	− 1.87	141.8	324.9	230.7	126.1	− 3.53	− 1.60
	Median	154.3	924.8	292.5	− 6.6	− 2.29	226.6	675.6	387.4	373.5	− 4.12	− 2.37
	Mean	198.6	821.6	323.4	− 8.2	− 2.53	247.2	724.8	393.9	351.9	− 4.34	− 2.53
	Max	403.0	1339	679.4	− 5.3	− 4.73	402.4	1154	577.0	583.8	− 6.14	− 3.97

*FC meant “femoral cortex”; SC meant “screw”; LP meant “long plate”; and SP meant “short plate”

### Von Mises stress distribution of the screws

**Fig. 5:**
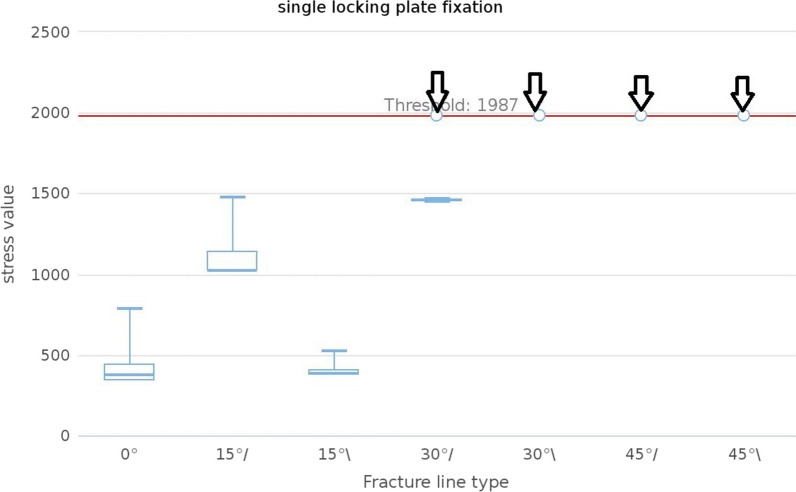
The median maximum and minimum values were shown in the plot. The black arrow meant that this group did not converge

**Fig. 6:**
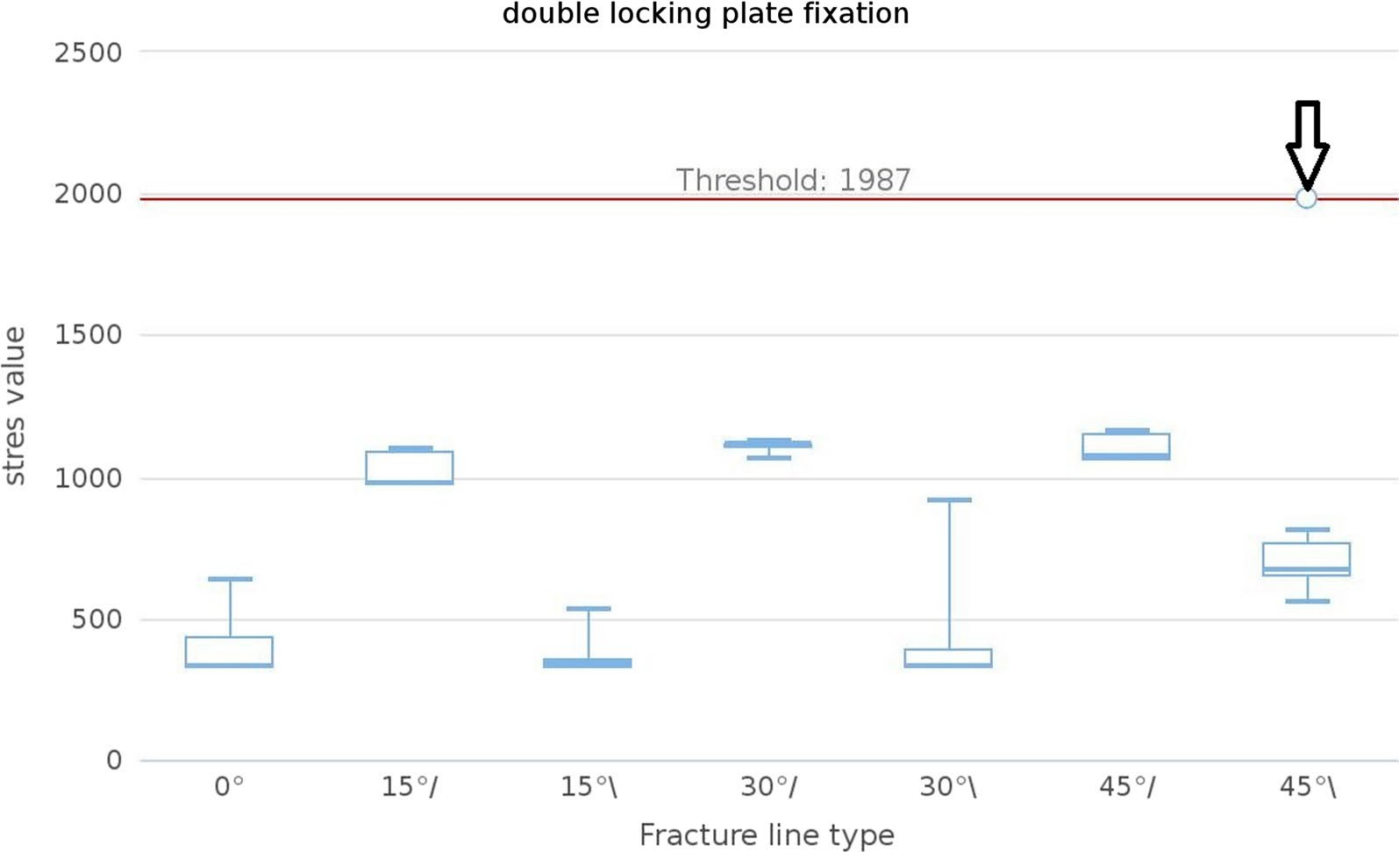
The median maximum and minimum values were shown in the plot. The black arrow meant that this group did not converge

In the simulation of all models, a significant stress concentration was observed at the area where the screw meets the cortical bone. Compared to the ULLP model, the maximum von Mises stress distribution at this position was much smaller for all the BLLP models (Fig. 4). The maximum stress in the screw of the 9H and 13H ULLP models were 779.5 MPa and 679.4 MPa, respectively. However, the maximum stress of the stress in the 9H and 13H BLLP models were 574.8 MPa and 577 MPa (Table 3). Furthermore, when the fracture line type was “/,” the maximum stress value of the screw was significantly higher than that of “\.” The number of convergences in the ULLP model group was significantly less than that of the BLLP model group, except for a non-convergence situation at “45°,” while all BLLP models were able to converge successfully (Figs. 5 and 6).

### Amount of displacement with the degree of bone defect or fracture line angle

**Fig. 7:**
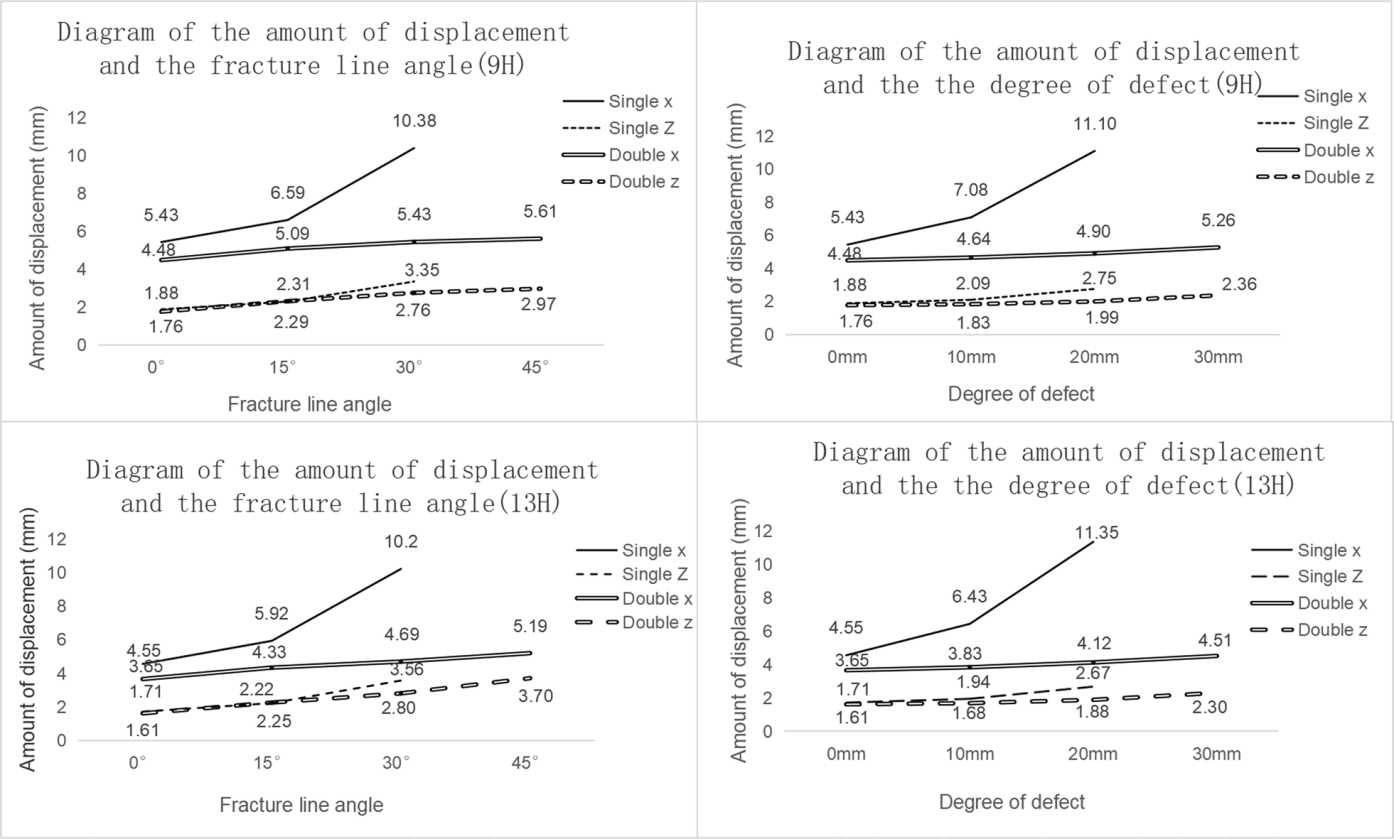
Fracture end displacement diagram

In the simulation of all models, the maximum displacement values (transverse and longitudinal) of all BLLP models were much smaller than those of the ULLP models (Fig. 7, Table 3). The maximum transverse displacement values (*X*) of the 9H and 13H ULLP models were − 26.56 mm and − 18.12 mm, respectively. The maximum lateral displacement (*X*) of 9H and 13H BLLP models were − 7.17 mm and − 6.14 mm, respectively. The maximum longitudinal displacement values (Z) of the 9H and 13H ULLP models were − 6.25 mm and − 4.73 mm, respectively. The maximum longitudinal displacement values (*Z*) of the 9H and 13H BLLP models were − 4.45 mm and − 3.97 mm (Table 3). Furthermore, when changing the bone defect, the displacement of the ULLP model was more than that of the BLLP model, and in the case of only changing the angle of the fracture line, except for the displacement value of the *z*-axis direction in the simulation of ULLP model in 15°, the displacement of the ULLP of the remaining models was greater than that of the BLLP.

## Discussion

In the reduction of OSFF, acquiring support from the bilateral cortex at the fracture site immediately after surgery is important. However, for UOSFF, particularly those with large bone defects and treated with ULLP, fixation failure or implant breakage is more likely to occur. This may be due to the loss of continuity of bilateral cortex in such fractures, the obvious loss of bone quality, and the inability to effectively support the distal femur, when the lateral locking plate is applied alone, the medial side cannot maintain the stability of the inner femur due to the lack of sufficient support, resulting in a significant increase in the displacement of the medial fracture, increasing the risk of delayed fracture union or bone disunion, and long-term concentration of stress on the lateral bone plate will eventually lead to internal fixation failure [20, 21]. Our study was based on the FE method and found that BLLP of the femur can significantly reduce the probability of internal fixation failure (the number of convergences of ULLP model was 30, while that for BLLP models was 153, each group with a total of 154 models), providing a biomechanical evidence for treating UOSFF with BLLP. Currently, biomechanical analysis of UOSFF and implants is common. The stress distribution can be analyzed using FE method, by setting parameters that reflect actual conditions, simulating the original tissue using discrete finite elements, and calculating the stress values within it. If the stress is too high, it will result in the destruction of bone tissue. The von Mises stress distribution of the implant is an indicator of metal yield strength. The higher the stress value on the implant, the higher the probability of implant failure [22–25].

The femoral cortical stress analysis showed that under the ULLP model, the number of coverage models in “/” group was significantly greater than that in “\” group, while under the BLLP model, convergences of “/” model was also greater than “\,” and there was only one case of non-convergence, distributed in the fracture line type of “\,” the angle of the fracture line was 45°, and the defect degree was 30 mm. However, the distribution of maximum stress in “/” group is generally larger than that in “\” group (*p* < 0.05). The following were the causes of these “abnormal phenomena”: (1) In the standing position, the transmission of human force is not vertical downward but biased outward. Under the condition of the fracture line type is “\,” the lower part of the femur can partly support the transmitted force, resulting in a difference in the maximum stress value. (2) The fixation of the long locking plate located on the lateral side plays a more supportive role than the model with the fracture line type “/” compared to the model with the fracture line type “\.” (3) The small locking plate on the medial side can share the stress of the long locking plate on the lateral side, but its stability is not as good as that of the long locking plate on the lateral side. (4) The degree of defect has a great impact on the stability of the model. The larger the femoral defect, the more unstable the model.

The position selection of the distal fixation screw is often limited by clinical conditions and cannot be in the correct position [34]. The distal screws and plates in this paper were locked in the most ideal and stable state after referring to the relevant literature (Fig. 2). From the stress analysis of the plates and screws, it was found that the maximum stress values of the plates were most likely to occur around the fracture line, while the screws were most likely to occur at the contact points with the bone cortex. This is consistent with what has been stated in many previous articles [12, 22–25]. Moreover, the stress values in the ULLP model were significantly higher than those in the BLLP model (*P* < 0.05). BLLP could significantly relieve the highly concentrated stress distribution caused by ULLP. The stress values increased with the increase in fracture line angle and bone defect degree. The ULLP model began to exhibit non-convergence when the fracture line type was “/,” and the fracture line angle was 30°. When the fracture line angle was 45°, the model did not converge at all. When the fracture line type was “\” and the fracture line angle was greater than 30°, the model did not converge at all. The BLLP models had only one non-converging case. For patients with a fracture line angle greater than 30° and accompanying bone defects, it is strongly recommended to use the BLLP internal fixation method instead of the ULLP internal fixation method. Additionally, surgeons should pay attention to the deformation of the screws at the contact points between the plate and bone cortex at the fracture line, and repair them in a timely manner.

The previous studies have found that shear movement (*x*-axis displacement) inhibits healing tissue formation, while proper longitudinal movement (*z*-axis displacement) promotes healing tissue formation [12]. In this study, we found a large displacement of the model in the x-axis direction when changing the degree of bone defect and a large displacement in the z-axis direction when changing the angle of the fracture line. Thus, we believe that if the fracture is accompanied by a large bone defect, attention should be paid to the formation of callus, and if the fracture line is large, more attention should be paid to the bearing of the plate. However, adding a medial plate can effectively reduce the longitudinal and shear movement of the femoral fracture site. We also found that the stress values on the femur and equivalent displacement values at the fracture site were significantly decreased in the 13H group compared to the 9H group, while the stress values on the screws were significantly higher in the 13H group than in the 9H group. However, the number of convergences in the 13H group was not significantly greater than that in the 9H group. These results suggest that a longer locking plate can disperse the stress concentration on the femur to a certain extent, thus avoiding excessive displacement and providing more stable support for the formation of callus [16, 26–29].

**Fig. 8:**
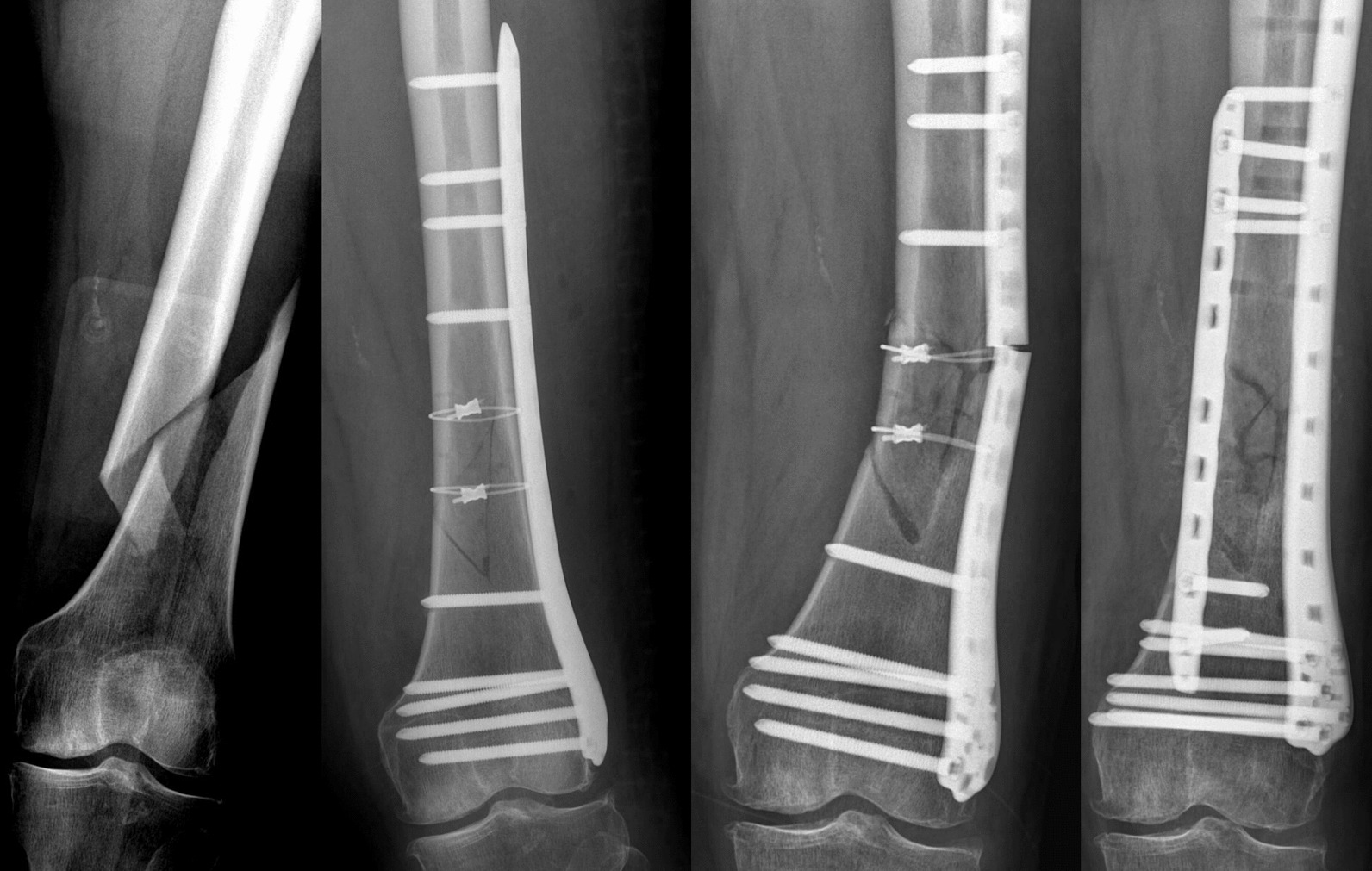
A typical case: (1) oblique fracture, (2) first internal fixation, (3) failed internal fixation, and (4) second internal fixation

The clinical validation of this paper came from a rare case in our hospital (Fig. 8), which was retrospectively analyzed. The angle of the fracture line was large (45°), and the fracture line type was “/,” the internal fixation (ULLP) was performed by an experienced orthopaedic surgeon. The operation was successful, there were no common complications such as infection and bone nonunion [35]. However, a sudden internal fixation rupture occurred 3 months after the operation during a normal walk. The reason for the failure of internal fixation was mainly because the ULLP could not be stabilized under this fracture after systematic evaluation, which was consistent with the concept proposed in this study.

This study has certain limitations, as it is not possible to simulate all situations in FE analysis. In our simulations, we did not consider patient-specific measurements of soft tissue thickness or muscle function. Moreover, there is no way to choose the right in vitro experiment to validate the results because of the sheer number of models.

## Conclusions

Our data demonstrate that more caution should be exercised in patients in the following four cases: First, when the angle of the fracture line is large (30°, 45°). Second, when the fracture line type is “/.” Third, when the bone defect is large. Fourth, when the bone defect is medial. To sum up, adding a small locking plate to the medial side not only effectively reduces the postoperative stress concentration distribution in the femur, but also reduces the longitudinal and shear movement at the fracture end, which is of great significance for the guidance of clinical treatment.

## Data Availability

The datasets generated during and/or analyzed during the current study are not publicly available, but are available from the corresponding author on reasonable request.

## References

[1] Cheung ZB, Nasser P, Iatridis JC, Forsh DA (2023). Orthogonal plating of distal femur fractures: a biomechanical comparison with plate-nail and parallel plating constructs. J Orthop.

[2] Bäumlein M, Klasan A, Klötzer C, Bockmann B, Eschbach D, Knobe M, Bliemel C (2020). Cement augmentation of an angular stable plate osteosynthesis for supracondylar femoral fractures—biomechanical investigation of a new fixation device. BMC Musculoskelet Disord.

[3] Bottlang M, Lesser M, Koerber J, Doornink J, von Rechenberg B, Augat P, Fitzpatrick DC, Madey SM, Marsh JL (2010). Far cortical locking can improve healing of fractures stabilized with locking plates. J Bone Joint Surg Am.

[4] Magill H, Ponugoti N, Selim A, Platt J (2021). Locked compression plating versus retrograde intramedullary nailing in the treatment of periprosthetic supracondylar knee fractures: a systematic review and meta-analysis. J Orthop Surg Res.

[5] Antao NA, Londhe S, Toor R, Shirishkar R, Aiyer S (2021). Short-term results of a novel management of supracondylar fracture with coexisting osteoarthritis with bifold fixation and total knee arthroplasty. Arthroplasty.

[6] Wright DJ, DeSanto DJ, McGarry MH, Lee TQ, Scolaro JA (2022). Nail diameter significantly impacts stability in combined plate-nail constructs used for fixation of supracondylar distal femur fractures. OTAI.

[7] Yamakawa Y, Masada Y, Okuda R, Matsumoto T, Uehara T, Yorimitsu M, Ozaki T (2023). Double plating via anterolateral and posterolateral approach for distal femoral fracture. Trauma Case Rep.

[8] Herrera A, Albareda J, Gabarre S, Ibarz E, Puértolas S, Mateo J, Gracia L (2020). Comparative analysis of the biomechanical behavior of anterograde/retrograde nailing in supracondylar femoral fractures. Injury.

[9] Metwaly RG, Zakaria ZM (2018). Single-incision double-plating approach in the management of isolated, closed osteoporotic distal femoral fractures. Geriatric Orthop Surg Rehabil.

[10] Wright DJ, Desanto DJ, Mcgarry MH (2020). Supplemental fixation of supracondylar distal femur fractures: a biomechanical comparison of dual-plate and plate-nail constructs. J Orthop Trauma.

[11] Chapman MW, Finkemeier CG (1999). Treatment of supracondylar nonunions of the femur with plate fixation and bone graft. J Bone Joint Surg Am.

[12] Habet N, Elkins J, Peindl R, Killen C, Lack WD (2019). Far cortical locking fixation of distal femur fractures is dominated by shear at clinically relevant bridge spans. J Orthop Trauma.

[13] Stewart MJ (1966). Fractures of the distal third of the femur. Bone Joint Surg.

[14] Bottlang M, Feist F (2011). Biomechanics of far cortical locking. Orthop Trauma.

[15] Wang BR, Jing LY, Ruan YP (2005). Double plate fixation of complex distal femoral fractures. Pract Med.

[16] Lujan TJ, Henderson CE, Madey SM, Fitzpatrick DC, Marsh JL, Bottlang M (2010). Locked plating of distal femur fractures leads to inconsistent and asymmetric callus formation. J Orthop Trauma.

[17] Stoffel K, Dieter U, Stachowiak G, Gächter A, Kuster MS (2003). Biomechanical testing of the LCP—how can stability in locked internal fixators be controlled?. Injury.

[18] Beingessner D, Moon E, Barei D, Morshed S (2011). Biomechanical analysis of the less invasive stabilization system for mechanically unstable fractures of the distal femur: comparison of titanium versus stainless steel and bicortical versus unicortical fixation. J Trauma.

[19] Anderson DD, Thomas TP, Campos Marin A, Elkins JM, Lack WD, Lacroix D (2014). Computational techniques for the assessment of fracture repair. Injury.

[20] Stoffel K, Dieter U, Stachowiak G, Gachter A, Kuster MS (2003). Biomechanical testing of the LCP—how can stability in locked internal fixators be controlled?. Injury.

[21] Bottlang M, Doornink J, Lujan TJ, Fitzpatrick DC, Marsh JL, Augat P, von Rechenberg B, Lesser M, Madey SM (2010). Effects of construct stiffness on healing of fractures stabilized with locking plates. J Bone Joint Surg Am.

[22] Hoffmann MF, Jones CB, Sietsema DL, Tornetta P, Koenig SJ (2013). Clinical outcomes of locked plating of distal femoral fractures in a retrospective cohort. J Orthop Surg Res.

[23] Goffin JM, Pankaj P, Simpson AH (2012). The importance of lag screw position for the stabilization of trochanteric fractures with a sliding hip screw: a subjectspecific finite element study. J Orthop Res.

[24] Yuan GX, Shen YH, Chen B, Zhang WB (2012). Biomechanical comparison of internal fixations in osteoporotic intertrochanteric fracture. A finite element analysis. Saudi Med J.

[25] Furui A, Terada N, Mito K (2018). Mechanical simulation study of postoperative displacement of trochanteric fractures using the finite element method. J Orthop Surg Res.

[26] Yamagishi M, Yoshimura Y (1955). The biomechanics of fracture healing. J Bone Joint Surg Am.

[27] Palomares KT, Gleason RE, Mason ZD, Cullinane DM, Einhorn TA, Gerstenfeld LC, Morgan EF (2009). Mechanical stimulation alters tissue differentiation and molecular expression during bone healing. J Orthop Res.

[28] Hayward LN, Morgan EF (2009). Assessment of a mechano-regulation theory of skeletal tissue differentiation in an in vivo model of mechanically induced cartilage formation. Biomech Model Mechanobiol.

[29] Elkins J, Marsh JL, Lujan T, Peindl R, Kellam J, Anderson DD, Lack W (2016). Motion predicts clinical callus formation. J Bone Joint Surg.

[30] Polikeit A, Nolte LP, Ferguson SJ (2003). The effect of cement augmentation on the load transfer in an osteoporotic functional spinal unit. Spine.

[31] Wu X, Yang M, Wu L, Niu W (2015). A biomechanical comparison of two intramedullary implants for subtrochanteric fracture in two healing stages: a finite element analysis. Appl Bion Biomech.

[32] Märdian S, Schaser KD, Duda GN, Heyland M (2015). Working length of locking plates determines interfragmentary movement in distal femur fractures under physiological loading. Clin Biomech.

[33] Akay M, Aslan N (1996). Numerical and experimental stress analysis of a polymeric composite hip joint prosthesis. J Biomed Mater Res.

[34] Nečas L, Hrubina M, Melišík M, Cibula Z, Olgun DZ, Horák Z (2019). Is Primary fixation with the sliding hip screw introduced into the non-ideal position sufficient for stable pertrochanteric fracture stabilisation? A biomechanical evaluation and experimental study. Period Polytech Mech Eng.

[35] Hrubina M, Skoták M, Krumpl O (2012). Osteosynthetic material breakage in patients treated with DHS for proximal femoral fracture. Rozhl Chir.

